# Evaluation of self-report adherence measures and their associations with detectable viral load among people living with HIV (PLHIV) in China

**DOI:** 10.1371/journal.pone.0203032

**Published:** 2018-08-30

**Authors:** Wendi Da, Xiaoming Li, Shan Qiao, Yuejiao Zhou, Zhiyong Shen

**Affiliations:** 1 Department of Health Promotion, Education, and Behavior, Arnold School of Public Health, University of South Carolina, Columbia, South Carolina, United States of America; 2 Guangxi Center for Disease Control and Prevention, Nanning, Guangxi, China; International AIDS Vaccine Initiative, UNITED STATES

## Abstract

**Objectives:**

Self-report antiretroviral therapy (ART) adherence has been consistently associated with clinical outcomes. This study aims to compare the accuracy of self-report ART adherence measures with varying recall timeframes or item contents to predict virological response.

**Methods:**

Data from a cross-sectional study among 2146 participants on ART in Guangxi, China were used. Detectable viral load was defined as viral load > 50 copies/ml. Adherence was measured using the number of days on which all doses were taken in the past month (i.e., the “one-month days taken” measure), the number of days on which any dose was missed in the past month (i.e., the “one-month days missed” measure), missed doses over the past 3 days, and missed days over the past weekend. Each adherence measure was dichotomized at an empirically determined cut-off to determine poor vs. good adherence. Accuracy of using each dichotomized adherence measure to predict detectable viral load was assessed by sensitivity, specificity, and the area under the receiver-operating characteristic (AUROC) curve. Logistic regressions were used to calculate the association between poor adherence and detectable viral load.

**Results:**

All four measures had sensitivity below 10.0%, specificity above 90.0%, and AUROC slightly above 0.50. In univariate logistic regression, detectable viral load was statistically significantly associated with poor adherence determined by the one-month days taken measure (OR = 1.98, 95% CI 1.15–3.42), the 3-day measure (OR = 2.18, 95% CI 1.10–4.34), and the weekend measure (OR = 2.86, 95% CI 1. 54–5.34). After adjusting for covariates, statistically significant association persisted only for the weekend measure (OR = 2.57, 95% CI 1.33–4.99).

**Conclusions:**

Adherence measures asking about days on which all doses were taken might work better than items asking about days on which respondents missed their doses, and weekend measures should be included to comprehensively capture adherence behaviors. Further studies looking at intermediate timeframes are also needed to capture patients’ dose-missing patterns that may better predict detectable viral load.

## Introduction

According to the 2015 China AIDS Response Progress Report, there were 501,000 reported people living with HIV (PLHIV) in China by the end of 2014 [1]. Access to antiretroviral therapy (ART) is rapidly expanding in these years, with the number of PLHIV who were currently receiving ART increasing from 126,448 in 2011 to 295,358 in 2014 nationwide [1]. In 2014, the Joint United Nations Programme on HIV and AIDS (UNAIDS) launched its ambitious 90-90-90 target for 2020, with the third target requiring 90% of patients on ART reaching viral suppression [2]. Although some countries are already remarkably close to achieving the third 90, China has been identified as one of the countries furthest from achieving this target in a 2016 meta-analysis of national HIV treatment programmes, with only 36% of PLHIV on ART achieving viral suppression (defined as VIRAL LOAD < 1000 copies/mL) [3]. Better virological responses were found by Huang et al. who followed PLHIV in Shenzhen, China over 10 years, where the rate of virological failure (defined as VIRAL LOAD ≥ 1000 copies/mL) was 6.7% at month 6 but increased to 31.5% at month 96 [4].

Poor ART adherence has been identified as one of the most important causes of virological failure, and once it occurs, improving adherence at that point may be too late with the possible emergence of drug-resistant virus [5]. Therefore, detecting and addressing poor ART adherence is a crucial step in the continuum of HIV care. Some clinical studies employed objective measures of ART adherence such as biomarkers and electronic monitoring devices [6]. The application of these measures, however, was limited in both clinical and research settings despite their high accuracy because of high cost and logistical complexities. Therefore, self-report still remained the most widely used adherence measure, which was found to be highly correlated with objective adherence measures [7–9]. However, there is a lack of consensus among both researchers and practitioners on the best self-report measure which often comes with different recall timeframes and item contents. Finding a relatively accurate and easy-to-implement self-report adherence measure, therefore, is important in monitoring and enhancing HIV treatment success as it provides an opportunity to intervene before clinical outcomes deteriorate.

One primary challenge related to self-report adherence measures is that these measures are prone to various biases such as social desirability and recall-error which could threat the internal validity [7]. Because of such challenge, the validity of self-report ART adherence measures has also been widely assessed using various criteria including virological response [8]. Most of the existing studies have assessed the correlation between ART adherence and virological response using single self-report measure with limited options of recall timeframes (e.g., recent 3-day, weekend, or one month), item contents (e.g., missed doses or missed days), and response tasks (e.g., frequencies of events, or numbers of events) [8]. Of limited studies comparing self-report adherence measures with different timeframes, many had small sample sizes, or assessed only convergent validity using device-based adherence as the criterion [10–13]. Studies comparing the predictive validity of self-report ART adherence measures with different recall timeframes and item contents using virological response as the criterion were even fewer.

The primary objective of the current study is to identify the optimal recall timeframe and item content of self-report ART adherence measures that best predict virological response. Utilizing cross-sectional data from a large representative sample of PLHIV in China, we compared the performance of four self-report ART adherence measures with different recall duration and item content in predicting detectable viral load by calculating sensitivity, specificity, and the area under the receiver-operating characteristic curve (AUROC). In addition, we assessed the association between the four self-report ART adherence measures and detectable viral load while controlling for potential sociodemographic, behavioral, and clinical covariates.

## Materials and methods

### Study design

Data in the current study were derived from a cross-sectional survey conducted from October 2012 to August 2013 in the Guangxi Autonomous Region (“Guangxi”) of China. Details of the study design have been published elsewhere [14]. Briefly, we selected 12 sites with the largest cumulative number of reported HIV/AIDS cases from 17 cities and 75 counties in Guangxi. The inclusion criteria were as follows: confirmed diagnosis of HIV infection; age 18 years or older; having the mental competence to provide informed consent and complete the survey. Approximately 10% of the patients at each site were randomly sampled, and a total of 3002 patients (about 90% of those contacted) agreed to participate. The survey was conducted in offices of local Center for Disease Control and Prevention (CDC) or HIV clinics where the participants received medical care. A total of 2987 participants completed the survey. Each participant received a small gift equivalent to five U.S dollars as a token of appreciation at completion of the survey.

### Ethical statement

The study protocol was reviewed and approved by the Institutional Review Boards at Wayne State University in the U.S and Guangxi CDC in China. All participants provided written informed consent prior to participation in the survey.

### Measures

#### Viral load

Participants’ most recent viral load data were retrieved from their medical records. Detectable viral load was defined as viral load > 50 copies/ml, a threshold used to determine virological failure in the 2011 China Guideline of Diagnosis and Treatment for AIDS [15].

#### ART adherence measures

Four self-report measures were used to assess patients’ ART adherence over the last month, the past 3-days, and the past weekend prior to the study visit. For the one-month days taken measure, participants were asked to report the number of days they took all doses of their HIV medication as prescribed. Percent adherence was calculated dividing the reported number of days by 30 days. For the one-month days missed measure, participants were asked to report the number of days on which they did not take any dose of their HIV medication. Percent adherence was calculated by first subtracting the reported number of days missed from 30 days and then dividing the result by 30 days. For the 3-day adherence measure, participants were asked to report the number of doses taken and the number of expected doses during the past three days. Percent adherence was calculated dividing doses taken by doses expected. For the weekend adherence measure, participants were asked whether they took their HIV medication on both, either, or neither days over the past weekend.

#### Covariates

Alcohol use was measured by the Alcohol Use Disorders Identification Test Consumption (AUDIT-C) questionnaire, and depression was measured by the 10-item Center for Epidemiologic Studies–Depression (CESD-10) scale [16,17]. Information on substance use and sociodemographic characteristics such as age, gender, ethnicity, education, household monthly income was also collected during the survey. Time on ART and most recent CD4 count were retrieved from medical record.

### Analysis

All analysis was conducted using Stata 13.0 (College Station, Texas). As viral load data were only available for some participants, we examined differences in sociodemographic, behavioral, and clinical characteristics between those with and without viral load data. T-tests (for continuous variables) and chi-square tests (for categorical or binary variables) were used in the bivariate analysis.

Further analysis was conducted among participants on ART for at least 6 months, had viral load data, and responded to at least one self-reported ART adherence questions (n = 1535). Detectable viral load (i.e., viral load > 50 copies/ml) was coded as 1 and undetectable viral load (i.e., viral load ≤ 50 copies/ml) was coded as 0. To empirically determine the cut-offs of percent adherence to define poor (i.e., percent adherence below the cut-off) vs. good adherence (i.e., percent adherence no less than the cut-off), we employed pre-defined cut-offs of 100%, 95%, 90%, 85%, and 80% for the three continuous adherence measures to see which cut-off would produce a dichotomized variable that statistically significantly correlated with detectable viral load. Cut-offs were calculated using the receiver operator curve (ROC) analysis in cases where none of the pre-defined cut-offs worked. For the ordinal weekend adherence measure, Fisher’s exact test (and post-hoc pair-wise comparisons with Bonferroni correction) was used to determine if there were significant differences in detectable viral load across groups. Kappa coefficients were calculated to examine the potential overlap among the four adherence measures.

Accuracy of using ART adherence measures (i.e, “the test”) to predict detectable viral load (i.e, “the disease”) was evaluated by calculating sensitivity, specificity, and area under the receiver operator curve (AUROC). Sensitivity was defined as the proportion of participants who self-reported as poorly adherent among those who had detectable viral load. Specificity was defined as the proportion of participants who self-reported as adherent among those who had undetectable viral load. Covariate-adjusted AUROC was also calculated.

Chi-square tests were used to examine the relationship between adherence and detectable viral load. Univariate logistic regression was used to calculate the odd ratio (OR) of using poor adherence to predict detectable viral load. Adjusted OR for poor adherence determined by each ART adherence measure was calculated with four separate multiple logistic regression models controlling for age, gender, substance use, alcohol use, depression, time on ART, and CD4 count.

## Results

**Fig 1:**
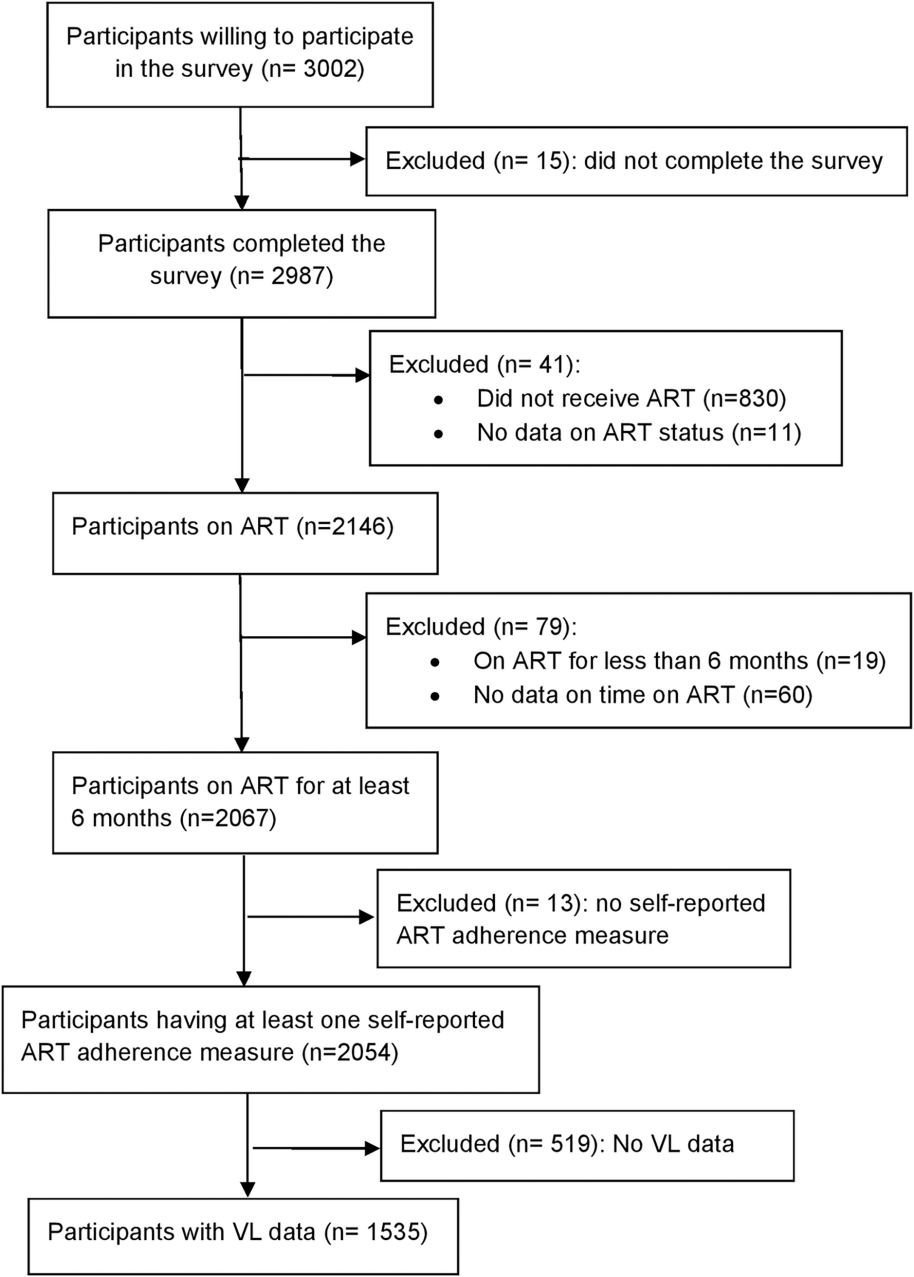
CONSORT flowchart of participants included in the study.

The flowchart of participants included in this study was shown in Fig 1. Sociodemographic, behavioral, and clinical characteristics of participants with (n = 1535) and without (n = 519) viral load data were shown in Table 1. The two groups were similar in terms of sociodemographic, behavioral, and clinical characteristics except gender, ethnicity, employment status, time on ART, CD4 count, and depression. Participants without viral load data were more likely to be males and of Han ethnicity but less likely to be employed. They also had shorter duration on ART, lower CD4 count, and higher depression scores.

**Table 1:** Demographical and clinical characteristics among participants receiving ART in Guangxi, China.

Variables	Participants with viral load data N = 1535^a^	Participants without viral load data N = 519^a^	P value
**Age (Mean±SD)**	42.5±12.3	42.2±13.0	0.604
**Male: n(%)**	915(59.6)	340(65.5)	0.017
**Han ethnicity: n(%)**	983(64.1)	427(82.3)	<0.001
**Education completed: n(%)**			
Elementary school	655(42.7)	240(46.6)	0.306
Middle school	658(42.9)	205(39.8)	
High school or higher	220(14.4)	70(13.6)	
**Employed: n(%)**	1142(74.6)	362(70.2)	0.046
**Monthly income < 1000 RMB: n(%)**	779(51.0)	279(54.4)	0.191
**Marital status: n(%)**			
Never married	123(8.2)	44(8.7)	0.062
Having a marital/live-in partner	1062(70.7)	377(74.8)	
Divorced/separated	122(8.1)	40(7.9)	
Widowed	195(13.0)	43(8.5)	
**With health insurance: n(%)**	1380(90.6)	465(90.5)	0.955
**Time on ART by month**			
Mean±SD	39.2±23.5	23.6±21.2	<0.001
Median (range)	34 (6–157)	15 (6–123)	<0.001
**CD4 cell count (cells/μl): n(%)**			
<350	885(57.6)	315(67.0)	0.001
350–499	365(23.8)	92(19.6)	
≥500	285(18.6)	63(13.4)	
**Alcohol use (Mean±SD)**	1.8±2.5	1.9±2.6	0.447
**Drug use: n(%)**	244(15.9)	93(18.0)	0.269
**Depression (Mean±SD)**	8.6±5.9	9.6±6.0	<0.001

^a^Not all the proportion was based on the entire sample due to missing data for some variables.

Among the 1535 participants who had viral load data, the mean age was 42.5 years old (SD = 12.3 years), and most were males (915/1535, 59.6%). Most (983/1533, 64.1%) were of Han ethnicity (the largest ethnic group in China), less than half (655/1533, 42.7%) completed elementary education only, most (1142/1530, 74.6%) were employed, and about half (779/1526, 51.0%) had a monthly household income less than 1000 RMB (Chinese currency, which equaled approximately 160 US dollars at the time of the survey). The majority (1062/1502, 70.7%) had a marital or live-in partner, and most (1380/1524, 90.5%) had health insurance. The average time on ART was 39.2 months (SD = 23.5), and more than half (885/1535, 57.6%) had CD4 counts less than or equal to 350 cells/mm^3^. The average AUDIT-C score was 1.8 (SD = 2.5), 15.9% (244/1531) participants had used drugs, and the average CESD-10 score was 8.6 (SD = 5.9).

### Cut-offs for determining poor vs. good adherence

The one-month days missed measure was not significantly correlated with detectable viral load across all five pre-defined cut-offs ranging from 100% to 80%. Therefore, we decided to use the cut-off at 88.3% determined by the ROC curve analysis. Poor adherence was determined as having a percent adherence lower than 88.3%.

The one-month days taken measure and detectable viral load were not significantly correlated until the pre-defined cut-off dropped to 90% (Chi-square = 7.46, p = 0.006). Therefore, poor adherence was determined as having a percent adherence lower than 90.0%.

The 3-day measure was significantly correlated with detectable viral load at the cut-off of 100% (Chi-square = 5.08, p = 0.024). Therefore, poor adherence was determined as having a percent adherence lower than 100%.

For the weekend measure, significant difference in detectable viral load was only found between those who reported no missing during the last weekend, and those who reported missing two days (Chi-square = 11.20, p = 0.001). Therefore, poor adherence was determined as missing any day during the weekend.

Kappa coefficient across the four measures were shown in Table 2, where low to medium concordance (0.14–0.34) was detected.

**Table 2:** Kappa coefficient across the four adherence measures.

Adherence measure	One-month days missed	One-month days taken	3-day	Weekend
**One-month days missed**	1	0.34	0.14	0.25
**One-month days taken**		1	0.29	0.32
**3-day**			1	0.26
**Weekend**				1

### Sensitivity, specificity, and AUROC

**Fig 2:**
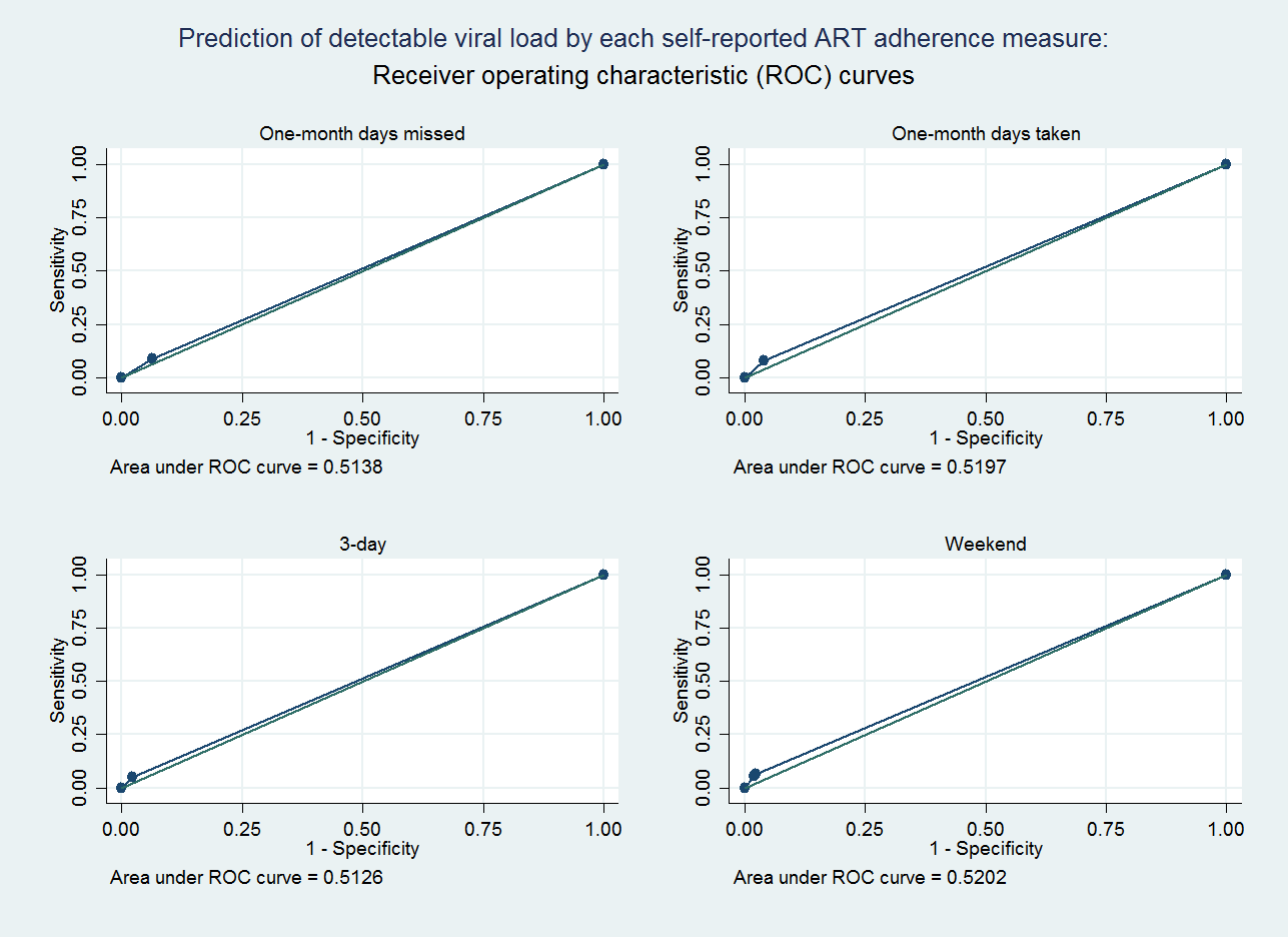
Prediction of detectable viral load by each self-reported ART adherence measure. Receiver operating characteristic (ROC) curves based on the univariate logistic regression model are shown.

**Fig 3:**
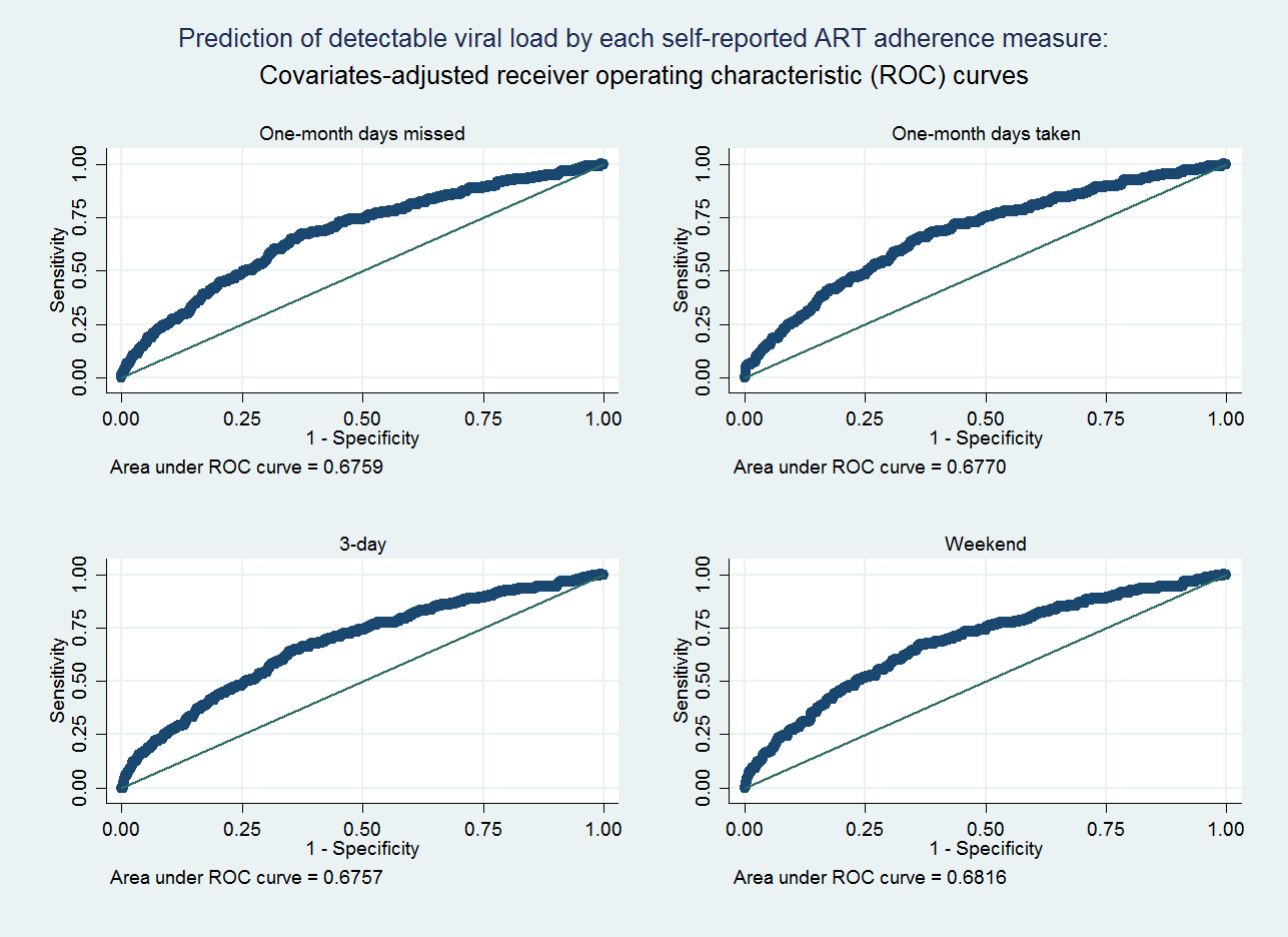
Prediction of detectable viral load by each self-reported ART adherence measure. Receiver operating characteristic (ROC) curves based on the multiple logistic regression model are shown.

Sensitivity, specificity, and AUROC of using adherence to predict detectable viral load were summarized in Table 3. All four measures had a high specificity, suggesting that reaching viral suppression was mostly related to good ART adherence. The sensitivity was below 10% for all four measures, suggesting that poor adherence was only a partial explanation for detectable viral load. The AUROC (Fig 2) for predicting detectable viral load ranged from 0.51 to 0.53, and were not significantly different across the four measures. After adjusting for covariates, the AUROC increased to 0.68 for all four measures (Fig 3).

**Table 3:** Predictive validity of adherence measures: sensitivity, specificity, AUROC, and odds ratio (OR).

Adherence measures	Detectable viral load			Sensitivity	Specificity	AUROC	Adjusted AUROC^a^	OR	AOR^a^
	Yes	No	P value						
**One-month days missed**									
Poor adherence	23(22.2)	81(77.9)	0.100	9.2(5.9, 13.4)	93.7(92.2, 95.0)	0.51(0.49, 0.53)	0.68(0.64, 0.71)	1.50(0.92, 2.43)	1.41(0.84, 2.37)
Good adherence	228(15.9)	1203(84.1)							
**One-month days taken**									
Poor adherence	19(27.1)	51(72.9)	0.012	7.6(4.7, 11.7)	96.0(94.8, 97.0)	0.52(0.50, 0.54)	0.68(0.64, 0.71)	1.98(1.15, 3.42)	1.75(0.98, 3.13)
Good adherence	230(15.8)	1225(84.2)							
**3-day**									
Poor adherence	12(29.3)	29(70.7)	0.023	4.9(2.6, 8.4)	97.7(96.7, 98.5)	0.51(0.50, 0.53)	0.68(0.64, 0.71)	2.18(1.10, 4.34)	2.04 (0.99, 4.18)
Good adherence	234(15.9)	1235(84.1)							
**Weekend**									
Poor adherence	16(34.8)	30(65.2)	0.001	6.5(3.8, 10.3)	97.6(96.6, 98.4)	0.52(0.50, 0.54)	0.68(0.64, 0.72)	2.86(1.54, 5.34)	2.57(1.33, 4.99)
Good adherence	231(15.7)	1240(84.3)							

^a^Adjusting for age, gender, time on ART, CD4, substance use, alcohol use, and depression.

### Logistic regression

The results of univariate and multivariate logistic regression models were shown in Table 3. In univariate regression, detectable viral load was statistically significantly associated with poor adherence determined by the one-month days taken measure (OR = 1.98, 95% CI 1.15–3.42), the 3-day measure (OR = 2.18, 95% CI 1.10–4.34), and the weekend measure (OR = 2.86, 95% CI 1. 54–5.34). After adjusting for covariates, statistically significant association persisted only for poor adherence determined by the weekend measure (OR = 2.57, 95% CI 1.33–4.99). Marginally significant associations were found for poor adherence determined by the one-month days taken measure (OR = 1.75, 95% CI 0.98–3.13) and the 3-day measure (OR = 2.04, 95% CI 0.99–4.18).

## Discussion

To the best of our knowledge, this study is one of the first efforts to empirically assess the accuracy of using self-report ART adherence to predict detectable viral load among PLHIV in China using a large representative sample. All four adherence measures had sensitivity below 10.0%, specificity above 90.0%, and AUROC slightly above 0.5. In univariate analysis, self-reported poor adherence determined either by the one-month days taken measure, the 3-day measure, or the weekend measure, increased the odds of having detectable viral load. No statistically significant association was found between the one-month days missed measure and detectable viral load. However, after adjusting for covariates, statistically significant association persisted only for the weekend measure.

### Sensitivity, specificity, and AUROC

Low sensitivity, high specificity, and low AUROC were detected in this study, which was consistent with previous studies assessing the validity of self-reported ART adherence measures to predict virological failure [18,19]. The high specificity for all four adherence measures suggested that good adherence is necessary to reach viral suppression, while the low sensitivity suggested that poor adherence was not the only factor that can be accountable for detectable viral load. Factors other than adherence may also influence virological response, including viral susceptibility, drug absorption and metabolism, potency of the regimen, as well as host immune status [20]. In addition, the improvement in the AUCs after adjusting for covariates suggested that the inclusion of potential confounders could improve the predictive validity of ART adherence measures.

### Differences in item content for the two one-month measures

For the one-month days taken measure, participants were asked about the number of days on which they took all the medication, while for the one-month days missed measure, participants were asked about the number of days on which they did not take any of their medication. Theoretically, responses to the two one-month measures should be highly correlated. However, only the one-month days taken measure was associated with detectable viral load in univariate logistic regression. One possible explanation was that, compared with the one-month days missed measure where participants were counted as “adherent” as long as they took their medication (not necessarily all doses as prescribed), the former employed a stricter criterion which better distinguished between good and poor adherence. Another explanation was that the behavior we asked participants to report differed between the two measures. As argued by Wilson et al., asking patients who are unintentionally nonadherent about missed doses may increase the risk that they will report an intention instead of action [21]. In our case, it is possible that participants’ responses to the “missed” question were biased towards underreporting their poor adherence behaviors.

### Optimal recall timeframe

Our results indicated that the one-month days taken measure might have similar accuracy compared with the 3-day measure as both were statistically significantly associated with detectable viral load in univariate logistic regression. After adjusting for covariates, marginally significant association was found for both measures. Although these two measures used different item contents (days versus doses), the one-month measure took dosing into consideration by applying a stricter criterion when defining adherence.

Longer timeframes may be less accurate because of its susceptibility to recall bias. Wilson et al. stated that patients cannot recall and enumerate specific pill-taking events and instead they answer adherence questions by estimating, which is often inaccurate [21]. However, Lu et al. argued that longer timeframes may capture the variation in adherence behaviors therefore is more clinically relevant [13]. Also, as participants in our study were recruited and assessed in the clinical settings where they received routine medical care, shorter timeframes could be more impacted by “white coat” effect where patients do better in adherence for the few days before the appointment [22]. Compared with the 3-day measure, the one-month measure was also less biased as a recall error for a single missed dose would have more weight with the short timeframe compared with the longer timeframe.

Ceiling effect is often a major concern for measuring socially desirable behaviors such as adherence. This type of measurement error results in a measure concentrating at the top of the scale with little variation to explain analytically [23]. In our study, the mean percent adherence did not vary much between the 3-day measure and the one-month days taken measure, but the one-month measure captured greater variation in adherence behaviors and identified more patients who self-reported as poorly adherent. Therefore, one-month recall timeframe was less susceptible to ceiling effect.

### The weekend measure

After adjusting for covariates, only the weekend measure was statistically significantly associated with detectable viral load. Therefore, we may infer that weekend measure has higher accuracy in predicting detectable viral load compared with the other three measures which may pick up both weekday and weekend adherence behaviors.

Disruption of daily routine has been identified as a major barrier to medication adherence, which often happens during the weekends when people take their “drug holidays” [24]. Considering the fluctuation of weekly adherence patterns as well as its health implications, Chesney et al. recommended asking about adherence during the last four days rather than only the last two days in the Adult AIDS Clinical Trials Group (AACTG) adherence instruments as this would increase the likelihood of including a weekend day [25]. Our study further supported the inclusion of weekend adherence behaviors when measuring ART adherence, which has critical implications for prevention and adherence interventions.

### Limitations

The study has several limitations. First, data were not available on viral load before ART initiation. Although we considered several important confounders of the relationship between adherence and detectable viral load (e.g., most recent CD4 count, substance use and depression), we did not have access to information on participants’ CD4 count or viral load prior to ART initiation, which are critical predictors of virological response. Second, information regarding laboratory procedures used to determine viral load was not available. Third, limited by space in the survey, we were not able to use the “ideal comparison” (i.e., identical format and different timeframes) to identify the optimal recall timeframe. It is possible that item content (e.g., doses versus days), not the recall timeframe, was responsible for the difference in accuracy. Finally, dosing frequency might modify the relationship between medication adherence and virological response, yet participants’ ART regimen information was not available.

## Conclusions

While it is widely agreed on the importance to promote adherence before virological failure and ART resistance mutations occur, questions remain regarding identifying accurate and feasible measure of ART adherence in clinical settings. This study compared the predictive validity of four self-report ART adherence measures calculated from either missed doses or missed days of varying timeframes with detectable viral load, yielding several key findings. First, we found that items asking about days on which all doses were taken might work better than items asking about days on which respondents missed their doses, especially for those who were unintentionally nonadherent. Second, in terms of recall timeframes, one-month recall can be of similar accuracy compared with 3-day recall, suggesting that one month can be a valid recall timeframe despite concerns for recall bias. Third, weekend adherence was most consistently associated with detectable viral load. As argued by Stirratt et al., determining the optimal recall timeframe requires balancing shorter intervals with longer intervals [7]. Further research comparing one month and weekend recall with intermediate timeframes (e.g., 7-day, 14-day) is needed to shed light on the weekly pattern of adherence behaviors and help optimize the selection of ART adherence intervention strategies.

In summary, our study suggests that the association between self-report measure of ART adherence and detectable viral load differs by recall timeframe and item content. Besides predictive validity, construct validity and convergent validity are also important, and we need further studies using cognitive interviewing and objective measures such as pill counts or Medication Event Monitoring System (MEMS) to best assess the overall validity of self-report measures.
